# An interview with Ambrosina Michelotti

**DOI:** 10.1590/2177-6709.23.2.022-029.int

**Published:** 2018

**Authors:** Ambrosina Michelotti

**Affiliations:** » Graduated in Dentistry (DDS) in 1984. » Specialist in Orthodontics (1991). » Professor in pregraduate and postgraduate courses in Orthodontics and TMD at the University of Naples Federico II. » Associate professor in Clinical Gnathology. » Responsible of the Master course on “Orofacial Pain and Temporomandibular Disorders” at the University of Naples Federico II. » Published more than 100 papers in Italian and international journals, and has lectured extensively around the world. » President of the European Academy of Craniomandibular Disorders (2010). » President of the Neuroscience group of IADR (International Association for Dental Research) (2011). » President of SIDA (Società Italiana Disfunzioni ed Algie Temporomandibolari; 2012-2013). » President of the RDC/TMD Consortium at the IADR (2013-2014). » Associate Editor of the European Journal of Oral Science. » Associate Editor of the Journal of Oral Rehabilitation. » Member of the editorial board of the European Journal of Orthodontics. »Referee of several national and international journals.

**Figure:**
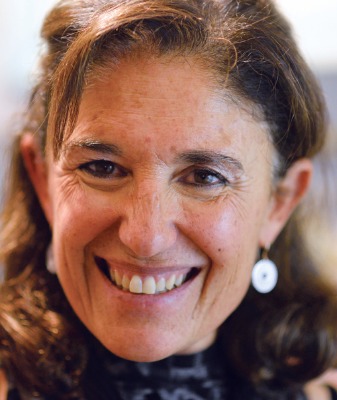

Professor Ambrosina Michelotti is one of the most important researchers in the field of Orthodontics, Temporomandibular Disorders and Orofacial Pain. I’m following her publications since I first read the revision article about the role of Orthodontics in Temporomandibular Disorders, published in 2010 on the Journal of Oral Rehabilitation, which helps to elucidate in a very didactic way the concepts based on scientific evidence that link these two areas. As I am involved on the same field of research, I became very enthusiastic of her work and competence. When in 2011 we had the opportunity to meet at a congress of the European Academy of Orofacial Pain, I had the chance to face another and even more important characteristic of Professor Ambrosina: her sympathy, happiness and cordiality. Since then, we have met in many congresses and recently, in last June, Professor Ambrosina attended the *Sociedade Brasileira de Disfunção Temporomandibular e Dor Orofacial* (SBDOF) meeting in São Paulo, where she lectured for more than 700 participants. I would like to highlight that Professor Ambrosina was one of the members of the team of professionals responsible for updating the Diagnostic Criteria for Temporomandibular Disorders (DC/TMD) last September in Newark, USA. And finally, I would like to thank the Dental Press Journal of Orthodontics for the opportunity of coordinating this interview, and also the professionals who contributed to this text. I am sure that DPJO readers will appreciate this interview, due to the broad scientific and clinical experience of this great lady of Orthodontics, TMD and Orofacial Pain. Enjoy it!

A Profa. Ambra Michelotti é uma das maiores pesquisadoras das áreas de Ortodontia, Disfunções Temporomandibulares e Dores Orofaciais. Acompanho de perto suas publicações, pois também estou envolvida nessas linhas de pesquisa, e tenho muita admiração pela sua competência. Seu artigo de revisão sobre o papel da Ortodontia nas Disfunções Temporomandibulares, publicado em 2010 no *Journal of Oral Rehabilitation*, esclarece de maneira muito didática os conceitos baseados em evidências científicas que permeiam essas duas áreas. Quando, em 2011, tive a oportunidade de ser apresentada a ela em um congresso da Academia Europeia de Dores Orofaciais, pude constatar que, além de inteligente e dedicada, sua simpatia e alegria se destacam. Desde então, foram vários encontros em congressos e, recentemente, em junho do ano passado, estivemos juntas no Encontro da Sociedade Brasileira de Disfunção Temporomandibular e Dor Orofacial (SBDOF), em São Paulo, onde presenciei sua aula, que foi muito aplaudida pelos mais de 700 participantes. Gostaria de destacar, também, que a Profa. Ambrosina participou, em setembro de 2017, como um dos integrantes da equipe de profissionais responsáveis pela atualização do Critério de Diagnóstico para a Disfunção Temporomandibular (CD/DTM) em Newark (EUA). Assim, gostaria de agradecer ao *Dental Press Journal of Orthodontics* pela grande oportunidade de coordenar essa entrevista, bem como aos professores que contribuíram com esse texto. Tenho certeza de que os leitores do DPJO vão aprender muito com a ampla experiência clínica e científica dessa grande dama da Ortodontia, DTM e Dores Orofaciais.

Ana Cláudia de Castro Ferreira Conti (interview coordinator/ coordenadora da entrevista)

In Brazil, Orthodontics and Temporomandibular Disorders/Orofacial Pain are two distinct specialties. How is the educational process of these two topics in Italy? How do you as an orthodontist divide your time between the two specialties? (Ana Cláudia Conti)

In Italy only Orthodontics is a specialty, and the post-graduated programs last three years. The educational process in Temporomandibular Disorders and Orofacial Pain includes knowledge acquired during the dental school program and master programs (usually lasting one year) for post-graduated education. The postgraduate programme in Orthodontics of the University of Naples Federico II is a full-time four years programme, with students treating both orthodontic and Temporomandibular Disorders and Orofacial Pain (TMD-OFP) patients, leading to the degree of Specialist in Orthodontics and of Master in TMD-OFP. I personally divide equally my time between orthodontics and TMD-OFP regarding research, teaching and clinic. From the clinical point of view, I treat both orthodontic and TMD-OFP patients following a separate path. If and when necessary, the management of patients is planned with a multidisciplinary team including the needed specialists. 

In your 2010 article published in the JOR, reviewing the role of Orthodontics in temporomandibular disorders (TMD),^1^ you questioned: “Does TMD cause malocclusion?”. In the answer, you pointed out three relatively uncommon and often aggressive pathologies that may affect temporomandibular joints (TMJ), namely: condylar hyperplasia, osteochondroma and rheumatoid arthritis. What is your opinion about the influence of more common TMJ pathologies, such as disc displacements occurring in the early stages of development, on child’s mandibular growth and symmetry? (Ricardo Tesch)

According to evidences coming from the literature, there are indications that disc displacements, with or without reduction, may either retard or arrest condylar growth and may thus be related to mandibular retrognathia and/or facial asymmetry. Indeed, in studies conducted in growing rabbits, it has been shown that experimentally induced unilateral or bilateral TMJ disc displacement can cause mandibular asymmetry or mandibular retrognathia. This seems to occur because non-reducing displacement of the TMJ disc during the growth period can induce histological reactions of the condylar cartilage in the animal model.^2^

Also in humans, TMJ disc abnormality was associated with reduced growth of mandible,^3^ and unilateral disk displacement without reduction has been associated to alteration of skeletal symmetry.^4^ However, the skeletal morphologies associated with TMJ disc displacement are not significantly different between symptomatic and asymptomatic patients. Hence, these skeletal consequences are unrelated with TMJ symptoms, being rare and small, and their clinical significance can be questioned.^5^

So, assuming that TMJ pathologies can influence mandibular growth, what are the characteristics of the patients in the orthodontic clinic that deserve further investigation? What are the basic aspects of TMJ clinical and imaging examination that orthodontists should consider? (Ricardo Tesch)

Before starting orthodontic treatment, it is advisable to always perform a screening examination for the presence of TMD. The gold standard for the diagnosis of TMD should be based primarily on information obtained from the patient’s history, a structured clinical examination and, in selective cases, TMJ imaging procedures. Indeed, in case of intracapsular dysfunction, TMJ noise by history and detected by the examiner are valid criteria for the diagnosis of disc displacement with reduction and degenerative joint disease. If needed, TMJ Magnetic Resonance Imaging is the reference standard for the diagnoses of disc displacements, and TMJ Computed Tomography Imaging is the reference standard for the diagnosis of degenerative joint disease. For medico-legal reasons, any findings, including TMJ sounds, deviation during mandibular movements or pain, should be recorded. However, imaging should not be used routinely, but rather considered when it is important to confirm the provisional clinical diagnosis for a specific patient. 

What are the precautions to be taken before, during and after orthodontic treatment in a patient with articular disc displacement with reduction? (Guilherme de Almeida) 

If the patient before the orthodontic treatment presents a joint disease, the first step is to make a differential diagnosis between clicking sounds, in order to determine whether it is a disc displacement with reduction (reciprocal click), a degenerative joint disease (crepitus), or a subluxation (eminence click). The second step depends on the specific diagnosis. Initial treatment remains a symptom-focused and behavioural treatment protocol. If the disc could be recaptured via intraoral appliance therapy, planned orthodontic treatment could be helpful to stabilize the position of the disc; however, both the recapture and the long-term stability of the recaptured disc cannot be guaranteed given the multifactorial aetiology of the pathology and the fluctuation of displaced discs. Hence, a treatment for disc recapture must be very carefully considered and the patient should be informed of the possibility of failure or relapse. If the diagnosis is acute disc displacement without reduction, the early intervention by ‘unlocking’ mandibular manipulation is the first therapeutic approach that can be attempted, and in case of successful and stable disc recapture, the protocol is like previously described. If the disc cannot be recaptured, mainly in case of chronic disc displacement without reduction, the symptom-focus and behavioural treatment protocol is continued, supplemented by joint distraction and heavy emphasis on treatment adherence. The goal of this stage is to transform the retrodiscal tissue into a pseudo-disc and to clinically assess the patient for pain, mandibular movements and reported function. If the patient presents with a subluxation, the symptom-focused and behavioural treatment approach is suggested and dental treatment can be initiated, recognizing that prolonged and excessive opening of the mouth should be avoided. In addition, the patient should be trained to avoid the subluxation movements, to control yawning and to perform stabilization exercises to control muscle incoordination. Finally, for degenerative joint disease, if the joints are not painful, restorative or orthodontic treatment can be immediately initiated, and the stabilization phase after dental treatment could include the use of an intraoral TMD appliance aiming to change the area of joint loading during sleep. If the joints are painful, a symptom-focused and behavioural treatment protocol together with pharmacotherapy is necessary until the patient is pain-free and stable. 

TMD signs and symptoms are fluctuating and unpredictable, and can emerge during dental treatment. At the beginning of dental treatment with a patient without TMD, the patient should be informed that because TMD characteristics are highly prevalent in the general population and its aetiology is multifactorial, it is not possible to establish any causal association between potential onset and dental therapy. If the patient subsequently presents signs or symptoms of TMD during active dental treatment, the first step is always to evaluate, as previously described, and make the differential diagnosis. The second step is to temporarily stop active orthodontic treatment to avoid exacerbating factors. The third step is to resolve the pain by following the conservative treatment protocol including pharmacotherapy, counselling, behavioural therapy, home exercises, and physical therapy. If required, an occlusal appliance can also be used to evaluate the interference-free position of the mandible. Afterwards, when the patient is pain-free, orthodontic treatment can be continued as previously planned or, if necessary, modified according to the patient’s condition, in order to obtain a stable occlusion.

If TMD signs and symptoms occur after the orthodontic treatment, the clinician should consider that the aetiology and the pathophysiology of TMD involve a large number of direct and indirect causal factors. Therefore, evaluation and treatment of TMD, whether it exists before, during, or after any dental treatment, require the same considerations as would be provided in the absence of any orthodontic treatment needs. It is important to rule out other causes of facial pain before investigating the teeth as the potential aetiological factor, because TMD is seldom the simple result of structural problem acting in isolation. 

The diagnosis of TMD had an important progress with the RDC/TMD validation project. In your opinion, is there any method/technique to be added in these criteria to better identify TMD patients? (Ana Cláudia Conti)

The clinical classification of temporomandibular disorders was published in 1992 by Dworkin and LeResche who proposed a dual-axes system, with axis I being the physical presentation of the problem, and axis II, the psychosocial and distress part of the problem for the patient. The Research Diagnostic Criteria for TMD (RDC/TMD) has now been updated to the Diagnostic Criteria for TMD (DC/TMD) maintaining the dual-axes approach, but providing reliable and valid criteria for common TMDs for clinical settings. In summary, the DC/TMD have advanced to an evidence-based system with greater validity for clinical use. However, the pathophysiology of the disorder remains poorly understood. Scientists have attempted to identify biomarkers and risk factors for a number of clinical TMD conditions. A biomarker generally refers to a measurable indicator of some biological state or condition. Although there may be many emerging biomarkers, it has been suggested that in particular bio-samples, functional brain imaging, structural imaging of tissues including the brain, quantitative sensory test and conditioned pain modulation would adequately qualify as potential biomarkers at present. Nevertheless, this is an emerging field that requires further research to be fully applied in the clinical setting. To date, the diagnostic criteria are mainly based on the information gathered from the history of the patient combined with the functional standardized clinical examination, supported when necessary by TMJ imaging.

A research from your group showed a positive influence from the mandibular plane inclination and the masseter thickness on the pain onset time and duration.^6^ In agreement, Bavia and Rodrigues Garcia^7^ showed that brachyfacial morphology influences the presence of painful TMD (P = 0.0077). Sonnesen and Svensson reported that subjects with deep bite may be more sensitive to glutamate-evoked pain and thermal stimuli,^8^ and that deep bite, in particular with retroclined upper incisors, can represent a risk factor for TMD.^9^ So I would like to ask if you believe craniofacial morphology may be the lost link between TMD and malocclusions. (Bruno Furquim)

The results of our previous study showed that short-faced subjects developed exercise pain earlier and endured less than normal- to long-faced subjects. We hypothesized that this finding might be related to differences in the composition of fiber types or to different mechanical advantage with short-faced subjects producing greater occlusal force, and higher intramuscular pressure, than normal- to long-faced subjects. Also, it has been shown that short-face subjects present more TMD pain. Finally, subjects with a deep bite are more sensitive to experimental pain, present more TMD or headache problems, and have a higher somatization score. 

Actually, the etiopathogenesis of temporomandibular disorders is multifactorial. Trauma, individual anatomic and neuromuscular abnormalities, biopsychosocial and neurobiological factors, adverse oral behaviors, and bruxism may contribute to their establishment. Muscle overload could be one possibility among the multiple factors that may be involved in the pathophysiology of pain sensitivity. Indeed, a significant association between daytime clenching and myofascial pain of the masticatory muscles was demonstrated, and the frequency of daytime clenching episodes is different between individuals suffering from myofascial pain and healthy pain-free controls. It could be hypothesized that short-face subjects performing parafunctional activities could overload the masticatory muscles more than the normal- to long-faced subjects. If this is the case, patients with a deep bite and with high somatization before orthodontic treatment might be hypervigilant to the occlusal changes, and the combination of parafunction and somatization could lead to more pain. However, this hypothesis should be confirmed by future studies.

Can fixed functional appliances used in adult patients cause any risk to TMJs and masticatory musculature? Would the intermaxillary elastics be capable of causing painful symptomatology? (Bruno Furquim)

Usually functional appliances are not indicated in adults since the aim of these appliances is to increase growth potential. An example of fixed functional appliance is the Herbst appliance, which is frequently used in the late adolescence and early adulthood. A longitudinal very-long-term follow-up study (up to 32 years) after Herbst therapy revealed only minor problems from the TMJ, and the TMJ findings 6 years and 32 years after Herbst treatment corresponded to those in the general population. Thus, in the very long term, this fixed functional appliance does not appear to be harmful to the TMJ. 

On the other hand, mandibular advancement devices (MAD), which are similar to functional appliances, are recommended for the treatment of sleep apnea. These appliances are worn during sleep-time for a very long period, lasting years, and may be associated with the development of symptoms of temporomandibular disorder (TMD) both for masticatory muscles and for TMJs. Therefore, when using MAD for obstructive sleep apnea (OSA), it is critical to perform a thorough assessment of the temporomandibular joint and associated structures, so that preexisting problems are identified and discussed with the patient. Also, if the oral appliance therapy is associated with increased pain in the temporomandibular complex in the initial period of use, given its transient nature, this pain is not a reason to contra-indicate a MAD, because TMDs appear limited with long-term oral appliance use.

Regarding the use of intermaxillary elastics, even though it can happen that patients could complain of TMD signs and symptoms, these are events that can be easily managed with a transient suspension of the active orthodontic treatment. According to the literature, in the long term, orthodontic treatment with bilateral Class II elastics does not cause significant orofacial pain or undesirable changes in the range of mouth opening. Therefore, this modality of orthodontic treatment cannot be considered responsible for inducing TMD.

Unilateral posterior crossbite in adults, well adapted and accompanied by acceptable facial asymmetry should be treated? (Guilherme de Almeida)

The orthodontic treatment in adults is indicated if the patient is motivated. Usually, the main reasons why an adult could seek orthodontic treatment are aesthetic or functional improvement. If the subject is well adapted to the unilateral posterior crossbite there is no functional indication. Also, if the asymmetry is the consequence of a skeletal impairment, it cannot be changed by the orthodontic treatment in adults. Furthermore, it should be considered that asymmetry dominates in the human body and that symmetry does not exist. Anatomical asymmetry of all body segments should thus be regarded as physiological variations of the ideal, which is a man-made construct.

Some TMD patients that also start an orthodontic treatment report an improvement of TMD signs and symptoms. These changes are usually interpreted as an occlusal influence (as an etiologic factor) in the TMD. How can you explain this relationship? (Daniela Gonçalves)

Across dentistry, numerous aetiological and therapeutic theories continue to be based on a presumed causal association between occlusion and TMD, and have justified the use of many occlusion-focused therapeutic approaches, including orthodontic treatment. This wide range of actively provided treatments serves to reinforce the validity of structural problems as cause for present or future TMD-related signs or symptoms. The remarkable responsiveness of TMD pain to a wide range of treatments, including placebo, means that mechanical interventions by the dentist aiming at “correcting” or intervening with a putative structural cause will likely be successful, at least in the short term. This short-term success thereby reinforces a belief that malocclusions are associated with the TMD correction. Long-term data, however, indicate that mechanically-oriented treatment does not insure prevention of future problems. Moreover, to assume that because a treatment of the mechanical type is successful therefore implies a mechanical cause for the symptoms is a logical fallacy. The research literature abundantly demonstrates the non-specific effects of almost every treatment provided to date for TMD, which has reinforced the strong hypothesis that TMD is largely a complex functional pain disorder, and the general treatment principles including behavioural therapy and home exercises are currently regarded as appropriate for the majority of patients with these complaints. 

You have a well-known research on the relationship between daytime clenching/tooth contacts and TMD pain. Could you tell us something about your latest findings? (Ana Cláudia Conti)

The role of daytime clenching in relation with TMD pain is widely discussed within the dental community. A significant association between daytime clenching and myofascial pain of the masticatory muscles has been demonstrated and the contributing role of oral parafunctions has been supported by the significant reduction of pain symptoms after habit reversal treatment. However, people are often unaware of their oral habits. We performed a study to measure the electromyographic activity of the masticatory muscles and to assess the frequency, amplitude, and duration of daytime clenching episodes in TMD patients with masticatory muscle pain, compared to a control group of pain-free individuals. We confirmed that individuals with masticatory muscle pain present a greater frequency of daytime clenching episodes than pain-free individuals.

In another study, we showed that application of an occlusal interference had a minor impact in individuals with low frequency of parafunctional behaviors, and aggravated masticatory muscle pain and headache in individuals with high frequency of parafunctions.

We also investigated the clenching episodes after the application of an occlusal interference in female with masticatory muscle pain. The results revealed that they did not avoid the interference and this is in contrast with the avoidance behavior recorded in pain-free female volunteers, who demonstrated a reduction of the masseter activity after insertion of an active interference, to skip the disturbing interference. The different reaction might be related to the fact that masticatory muscle pain patients have the habit of holding the teeth in contact more often than healthy individuals during wakefulness. The lack of adaptation supports the hypothesis that subjects with TMD could adapt less well to the introduction of an active occlusal interference than subjects without a TMD.

Finally, in another study, after the application of orthodontic separators, individuals with high somatosensory amplification and trait anxiety reported greater occlusal discomfort and pain than individuals with low somatosensory amplification and trait anxiety. This finding suggests an occlusal hypervigilance that is characterized by a persistent heightened attention on weak and infrequent sensations, and by an increased focus on somatic sensations interpreted as potentially more alarming, threatening, and disturbing. 

In conclusion, orthodontic practitioners should be aware of the combination of oral parafunction, TMD and psychological characteristics of their patients, and possibly should recognize those individuals who may be at risk for complications during irreversible prolonged dental and orthodontic treatments.

What is your opinion about the use of occlusal splint in children and adolescents? Which are the parameters of age and dental development to securely use this therapeutic approach? (Daniela Gonçalves)

The indications for using an occlusal splint in children and adolescents can be summarized in the following main conditions: 

1. It is indicated in children/adolescents with signs and symptoms of TMD. Indeed, TMD is quite common in adolescents, more prevalent in girls, with a prevalence of approximately 8%. In general, occlusal appliance therapy and counseling are the most commonly used treatment modalities for TMD among adolescents. The findings of a recently published study showed that adolescents with TMD pain treated with occlusal splint reported better pain relief compared to those treated with relaxation training^10^. 

2. In children/adolescents that present sleep bruxism, where the soft appliance is as common as the stabilization appliance. The main reason why the soft appliance is used more frequently is to avoid hampering the development of the jaws and teeth in children that are still growing, and to give an appliance that is cheaper than a stabilization appliance, and can be changed more easily. 

3. Occlusal splint is recommended in growing subjects affected by juvenile rheumatoid arthritis, with the purpose of increasing function of the joint and ensuring continuous growth of the mandible. Indeed, occlusal splint therapy changes and reduces excessive loading on the TMJ, causing less bone resorption.^11^

4. In children that had unilateral or bilateral condylar fracture. In this case, the conservative treatment has a satisfactory clinical outcome in restoring function and treating condylar fracture in children and adolescents.

What is your opinion about the use of botulinum toxin in the treatment of myofascial TMD? (Bruno Furquim)

Botulinum toxin is approved by Federal Drug Association (FDA) for the treatment of chronic migraine, cervical dystonia, axillary hyperhidrosis, adult upper limb spasticity, strabismus and blepharospasm associated with dystonia. The effect of the toxin terminates within 2-6 months. Often, the toxin has an analgesic effect that occurs earlier and lasts longer than its effect on muscle tone. However, evidence of an effect by botulinum toxins is still lacking for most pain conditions. A randomized clinical trial, evaluating the efficacy of botulinum toxin type A in patients with persistent myofascial temporomandibular disorders, did not find any significant changes after treatment as compared to saline injection.^12^ Also, a Cochrane review concluded that there is no evidence to support the use of botulinum toxin in the treatment of myofascial TMD.^13^
